# The pharmacology, pharmacokinetics, and toxicity of spinosin: A mini review

**DOI:** 10.3389/fphar.2022.938395

**Published:** 2022-09-12

**Authors:** Xiaolan Kuang, Ganshu She, Ting Ma, Wanna Cai, Jingjing Zhao, Bo Liu, Fangfang Xu

**Affiliations:** ^1^ Guangdong Provincial Key Laboratory of Clinical Research on Traditional Chinese Medicine Syndrome, Guangzhou Key Laboratory of Chirality Research on Active Components of Traditional Chinese Medicine, The Second Clinical College of Guangzhou University of Chinese Medicine, Guangzhou, China; ^2^ Department of Pharmacy, The Second Affiliated Hospital of Guangzhou University of Chinese Medicine, Guangzhou, China; ^3^ Department of pharmacy, GuangDong Women and Children Hospital, Guangzhou, China

**Keywords:** spinosin, pharmacology, pharmacokinetics, toxicity, *Ziziphus jujuba* Mill*.* var. *spinosa*

## Abstract

Spinosin, a natural flavone-*C*-glycoside that is mainly isolated from the seeds of *Ziziphus jujuba* Mill*.* var. *spinosa*. It exerts the effects to ameliorate the neurological disorders, such as hypnosis effects, improvement of cognitive function, sedation effects, and anxiolytic effects, as well as anti-melanogenic effect, cardioprotective effects, and anti-cancer activity. However, the insufficient basic research, unclear mechanisms, and poor bioavailability may limit the prospects of spinosin in clinical utilization. In this review, we comprehensively summarized the latest information on the pharmacology, pharmacokinetics, toxicity, and NMR characteristic of spinosin, to evaluate its potential therapeutic for clinical application, hoping to provide some rational perspective for the innovative agent development and usage of spinosin in future.

## Introduction

**FIGURE 1:**
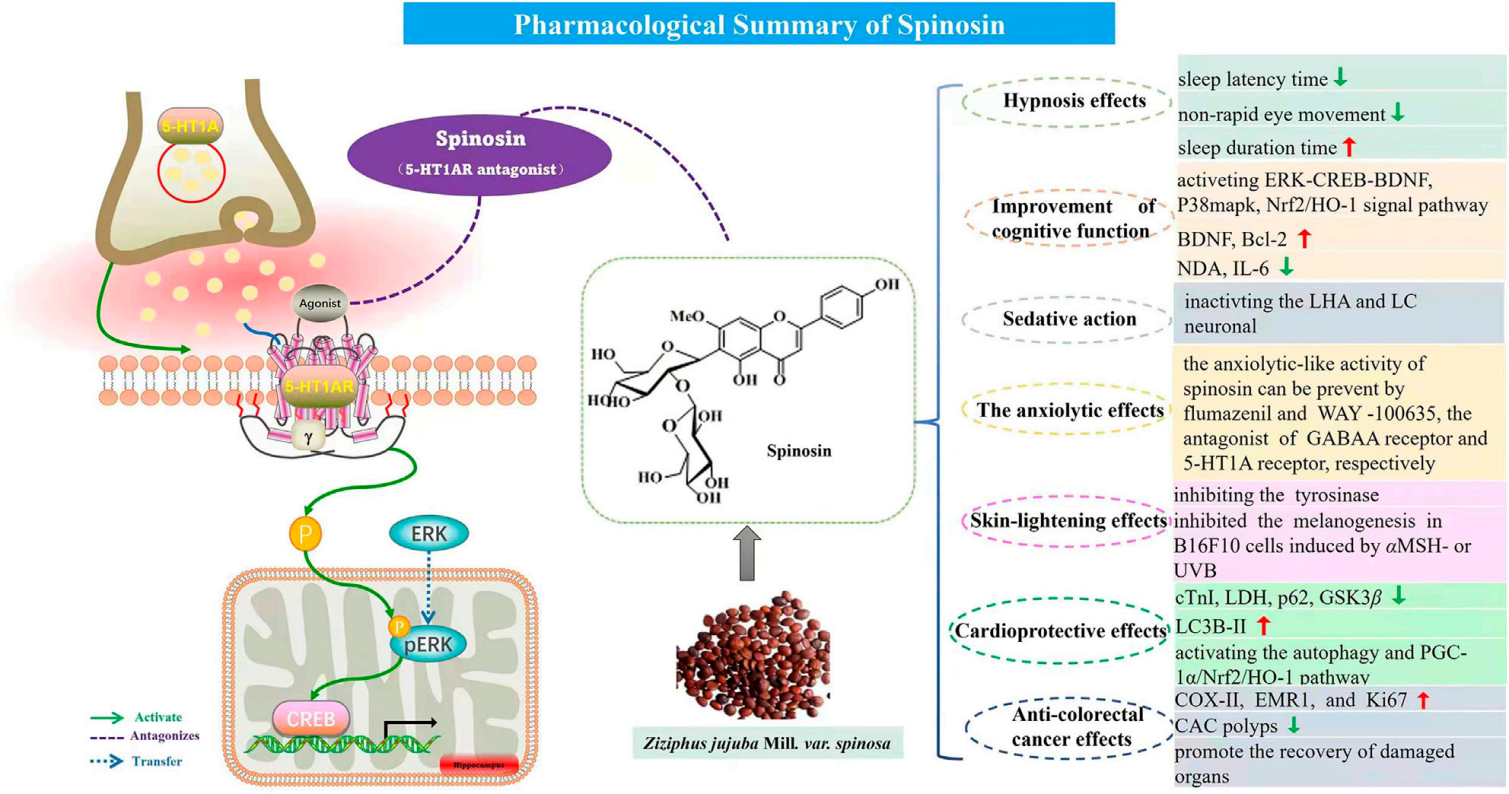
Pharmacological summary of spinosin.

Spinosin, (PubChem CID: 24771055, CAS No.: 72,063-39-9, MW: 608.5 g/mol), with the molecular formula of C_28_H_32_O_15_, is a natural flavone-*C*-glycoside that mainly exists in dried and ripe seeds of *Ziziphus jujuba* Mill*.* var. *spinosa* (ZJS) (Figure 1) (Wu et al., 2011). Recently, spinosin has also been isolated from *Cayaponia tayuya* (Vell.) Cogn (Aquila et al., 2009), *Passiflora edulis* Sims (Zucolotto et al., 2009), *Leonurus japonicus* Houtt. (Liu et al., 2018), and so on.

In traditional Chinese medicine (TCM), ZJS is sweet and sour in flavor, neutral in nature, and belongs to the liver, gallbladder, and heart meridians (Chinese pharmacopoeia, 2020). ZJS possesses the functions of nourishing the heart and liver, calming the mind and nerves, and condensing sweat and producing fluid that has been widely used for hypnosis, palpitations, dreams, sweating, and thirst (Chinese pharmacopoeia, 2020). It also has been widely used as an herb in the preparations of Chinese material medica to treat insomnia and anxiety for its main sedative and hypnotic effects. ZJS contains several groups of bioactive components including flavonoids, saponins, alkaloids, and fatty acids (Jiao et al., 2017), and saponins and flavonoids are the main effective components of sedation and hypnosis. Jujuboside A, jujuboside B, and spinosin are major components of total saponins and total flavones, respectively (Zhu et al., 2015). Spinosin is a *C*-glycoside flavonoid isolated from the ethanol extract of ZJS’s dried seeds (Huang et al., 2014), as one of the main bioactive components of ZJS, numerous explorations of the pharmacological effects of it have been reported, such as hypnosis effects (Wang et al., 2008, 2010, 2012; Wang et al., 2016a), improvement of cognitive function (Jung et al., 2014; Ko et al., 2015; Xu et al., 2020), anxiolytic effect (Wang and Yan, 2022), inhibition of melanin synthesis (Moon et al., 2019), and antioxidant effects (Zhang et al., 2020a).

Pharmacology research is crucial for the efficient and rational development of drugs, such as elucidating their mechanism, developing new usage of existing drugs, improving their efficacy, and reducing the toxicity of the medicine. The pharmacokinetic (PK) behavior of drugs plays an essential role in their pharmacological actions. The alterations of pharmacokinetic parameters may affect the drug’s therapeutic efficacy (Ma et al., 2012). Detailed toxicological data are the basis for risk assessment. The use of accurate and reliable toxicological data is the first step in hazard identification (Qu and Song, 2021). To better utilize the medicinal resource of spinosin, we discussed the research on spinosin in various research fields in the recent years covering its pharmacology, pharmacokinetics, and toxicology in this review.

## Pharmacology

### Hypnosis effects

The seeds of ZJS are used as a traditional herbal drug for the treatment of insomnia (Tsai et al., 2019). The potentiating sleep effect of spinosin was investigated in pentobarbital-treated (45 mg/kg, i.p.) mice. The results showed that pretreatment with spinosin (10 and 15 mg/kg) enhanced hypnotic effects, while using spinosin alone did not work. Further studies found that co-administration of spinosin (5 mg kg, p.o.) and 5-HTP (2.5 mg kg, i.p.) significantly reduced sleep latency and lengthened the sleep duration time (Wang et al., 2008). The aforementioned research suggested the regulation effect of the 5-HT system in pentobarbital-induced sleep.

The 5-HT1A receptor, a subtype of 5-HT receptors, plays an important role in the modulation of sleep and wakefulness. A deeper study revealed that spinosin (15 mg/kg, i.g.) lengthened the REM sleep time and increased the slow-wave sleep (SWS) mode in rats. A 5-HT_1A_ antagonist, the *p*-MPPI, reduced sleep latency and increased total sleep time and NREM sleep time. Conversely, a 5-HT_1A_ receptor agonist, the 8-OH-DPAT, reduced the NREM sleep, REM sleep, and SWS time in pentobarbital-treated rats. Spinosin could reverse the 8-OH-DPAT-induced reductions in the aforementioned sleep periods, suggesting spinosin may serve as an antagonist on the postsynaptic 5-HT_1A_ receptors (Wang et al., 2010). Further studies showed spinosin could potentiate pentobarbital-induced loss of righting reflex (LORR) in mice, and verified the antagonist of spinosin on presynaptic 5-HT_1A_ autoreceptor (Wang et al., 2012). Another research indicated that spinosin (20 mg/kg, i.p.) can increase the non-rapid eye movement (NREM) time and shorten the sleep latency time in the active phase of mice by antagonizing the 5-HT_1A_ receptor (Wang et al., 2016a).

Those results indicated that spinosin is an inhibitor on both somatodendritic 5-HT_1A_ autoreceptors and postsynaptic 5-HT_1A_ heteroreceptors, and this ingredient may be used as a potential drug for the treatment of hypnosis.

### Improvement of cognitive function

Alzheimer’s disease (AD) is a neurodegenerative disease with the characteristics of memory deterioration, cognitive function reduction, and behavioral impairments (Chu et al., 2012). The hallmark of AD is hippocampal synaptic dysfunction, which is becoming a potential target for the AD therapy.

Spinosin exerted the neuroprotective effects on cholinergic blockade-induced memory impairment in mice by extending the latency time in the passive avoidance task, lengthening the swimming time, and increasing the expression levels of phosphorylated extracellular signal-regulated kinases and cAMP response element-binding proteins in the hippocampus (Jung et al., 2014). Further research revealed that spinosin increased the number of immature neurons in the dentate gyrus region of the hippocampus and the neuronal cell propagation, and stimulated the neurons’ differentiation by the ERK-CREB-BDNF signaling pathway. The results indicated that spinosin can be used for the treatment of neurological cognitive dysfunction or psychiatric disorders (Lee et al., 2016).

The amyloid-*β*_1-42_ (A*β*_1-42_)–induced mouse model was used to evaluate the activities and mechanisms of spinosin in the treatment of AD. Spinosin improves the memory impairment induced by amyloid A*β*_(1–42)_ oligomer in mice verified by the passive avoidance task and the Y-maze task, decreasing the GFAP or OX-42 in the hippocampus, reducing the number of activated microglia and astrocytes, and enhancing the choline acetyltransferase (ChAT) expression (Ko et al*.*, 2015). Spinosin attenuated the long-term potentiation (LTP), which is the indicator that reflects learning and memory, by the improved plasmin level in hippocampi of 5XFAD mice induced by A*β* (Cai et al., 2020).

Numerous studies have revealed that neuroinflammation play a critical role in the occurrence and development of AD. Anti-inflammation has been considered to be one of the significant ways to improve or even treat AD. Spinosin has been reported to alleviate cognitive impairment by improving the neurotrophic factor (BDNF) and Bcl-2, decreasing the level of MDA, and inhibiting the inflammatory factor IL-6 in the brain (Xu et al., 2019). Another research showed that spinosin inhibited the expression of COX-2 and Bax protein caused by A*β*_25-35_, and improved the proportion of LTP. The conclusion suggested the repairment of spinosin on the learning and memory impairment induced by A*β*_25-35_ was mainly by inhibiting the inflammatory response (Du, 2021).

The oxidative stress is often accompanied by the occurrence of AD (Cervellati et al., 2016). Reactive oxygen species (ROS), including hydrogen peroxide (H_2_O_2_), hydroxyl radical (OH•), and superoxide anions (O_2_), is the major source of oxidative stress, which contributes to proteins, lipids, and DNA oxidation in brain tissues. p38MAPK is sensitive to stressful stimuli such as ROS and is related to the development process of AD. Spinosin showed the inhibitory effect on the intracellular ROS production induced by H_2_O_2_ in N2a cells. The in-depth mechanism research indicated spinosin inhibited A*β*_(1–42)_ production, decreased Tau phosphorylation, and improved synaptic structural plasticity induced by H_2_O_2_ through inhibiting the p38MAPK activation (Xu et al., 2020).

In addition, spinosin inhibited the production and accumulation of A*β*_1-42_ through influencing the amyloid precursor protein (APP) parade, by activating the antioxidative Nrf2/HO-1 pathway (Zhang et al., 2020c).

### Sedative action

The sedative activities of spinosin were evaluated by the climbing test and caffeine-induced hyperactivity model in mice. The results revealed that the number of mice that could not climb the ladder was increased and the frequency of crossing the hole was decreased after spinosin (500 and 1,000 mg/kg) was given (i.p.) (Shin et al., 1978; Shin et al., 1981). Spinosin was injected into mice (15 mg/kg, i.p.), and then 90 min later their brains were isolated and used for immunohistochemical analysis. The results revealed that spinosin markedly decreased c-Fos expression in the lateral hypothalamic area (LHA) and locus coeruleus (LC), suggesting that inactivation of the LHA and LC neuronal was the mechanism of spinosin on sedation (Zhang et al., 2020a).

### The anxiolytic effects

The anxiety disorder is one of the most common psychiatric disorders that affect the health of the general population. The anxiolytic effects of spinosin were evaluated by a plus maze, light/dark box test, and open field test in mice (Gu et al., 2018). Also, the results showed that spinosin exhibited the anxiolytic effects without influencing the spontaneous activity. Further research illustrated that the anxiolytic-like activity of spinosin can be prevented by flumazenil and WAY-100635, the antagonist of GABA_A_ receptor and 5-HT_1A_ receptor, respectively, which suggested its targets maybe GABA_A_ and 5-HT_1A_ receptors (Liu et al., 2015).

### Skin-lightening effects

As tyrosinase is the key factor in melanin production, inhibiting the tyrosinase can be used for skin pigmentation. Spinosin showed tyrosinase inhibitory activity (IC_50_ = 47 μM) and anti-melanogenesis effect (10, 20 μM) in B16F10 cells induced by *α*MSH- or UVB. The protein docking analysis further demonstrated that spinosin repressed the tyrosinase activity through the hydrogen bonds (Moon et al., 2019). This evidence suggested the potential of spinosin developed as a candidate for skin-lightening cosmetics.

### Cardioprotective effects

The acute myocardial infarction (AMI) rat model was used to investigate the cardioprotective effects of spinosin and its analog 6‴-feruloyl-spinosin. As a result, pretreatment with spinosin lessened myocardial tissue injury, reduced the serum levels of cTnI and LDH, and promoted autophagy by increasing LC3B-II levels in AMI rats. The mechanism data suggested that spinosin worked by inhibiting the GSK3*β*, and activating the autophagy and the activity of the PGC-1*α*/Nrf2/HO-1 pathway (Gu et al*.*, 2019).

### Anti-colorectal cancer effects

The polyphenol extraction from ZJS (ZJSP, 0, 50, 100, 150, and 200 μg/ml) exhibited anti-colorectal cancer (CRC) activity by inhibiting HCT-116 cell growth, increasing cell apoptosis in the HCT-8 and HCT-116 cells, and enhancing the sensitivity of HCT-8FU cells for 5-FU. The AOM/DSS-induced CAC mice were used to evaluate the CRC effect and the results showed the ZJSP (100 and 200 mg/kg) reduced the CAC polyps, promoted the recovery of damaged organs (heart, liver, spleen, lung, kidney, and pancreas), and raised the early CRC markers (COX-II, EMR1, and Ki67) in CAC mice. Further isolation and RP-HPLC-MS/MS results indicated spinosin was the anti-CRC constituent in ZJSP (Shan et al., 2020). This study indicated the potential usage of spinosin as a natural agent against CRC. The details pharmacological activities of spinosin are depicted in Table 1 and Figure 1.

**TABLE 1:** Pharmacology of spinosin.

Pharmacological effect	Detail	Cell line/model	Dose	Application	Reference
Hypnosis effects	Increase sleep time and reduce sleep latency assessed with the loss-of-righting reflex	Male ICR mice	0.1 ml/10 g	*In vivo*	Wang et al. (2008)
	Reduce sleep latency and increase total sleep time, slow-wave sleep (SWS) sleep time, and REM sleep time as inhibitor of postsynaptic 5-HT1A receptors	Male SD rats	5, 10, 15 mg/kg	*In vivo*	Wang et al. (2010)
	Potentiate pentobarbital-induced loss of righting reflex (LORR) in mice	ICR male mice	5, 15 mg/kg	*In vivo*	Wang et al. (2012)
	Increase non-rapid eye movement (NREM) time and shorten the sleep latency time in the active phase of mice	C57BL/6J mice	5, 10, 20 mg/kg	*In vivo*	Wang et al., 2016a
Improvement of cognitive function	Exert the neuroprotective effects on cholinergic blockade-induced memory impairment in mice by extending the latency time in the passive avoidance task, prolonging the swimming time, increasing the expression levels of phosphorylated extracellular signal-regulated kinases, and cAMP response element-binding proteins in the hippocampus	Male ICR mice	2.5, 5, 10, or 20 mg/kg	*In vivo*	Jung et al. (2014)
	Improve the memory impairment induced by amyloid A*β*_(1–42)_ oligomer in mice though the passive avoidance task and the Y-maze task, reduce the number of activated microglia and astrocytes, enhance the choline acetyltransferase expression	Male ICR mice	5, 10, 20 mg/kg	*In vivo*	Ko et al. (2015)
	Increase the proliferation and survival of neuronal cells and the number of immature neurons in the hippocampal dentate gyrus region, stimulate the differentiation of newly generated cells into mature neurons by activating of the ERK-CREB-BDNF signaling pathway	Male ICR mice	1.25, 2.5, 5, or 10 mg/kg	*In vivo*	Lee et al. (2016)
	Alleviate the cognitive impairment by decreasing the level of MDA and A*β*_(1–42)_ accumulation in hippocampus., improve the neurotrophic factor (BDNF) and B-cell lymphoma-2 (Bcl-2) in the brain, and inhibit the inflammatory response in the brain	Male-specific pathogen-free KM mice	10, 100 *μ*g/kg	*In vivo*	Xu et al. (2019)
	Attenuate amyloid *β* -induced long-term potentiation (LTP) impairment, and improve plasmin activity and protein level in the hippocampi of 5XFAD mice	ICR mice	3, 10, 30 μM	*In vivo*	Cai et al. (2020)
		Male 5XFAD mice			
	Prevent H_2_O_2_-induced oxidative damage via inhibiting A*β*_(1–42)_ production, decrease Tau phosphorylation, and improve synaptic structural plasticity though inhibition of p38MAPK.	N2a cell	25 μM	*In vitro*	Xu et al. (2020)
	Reduce A*β*_1-42_ production by activating the Nrf2/HO-1 pathway in N2a/WT and N2a/APP695 cells	N2a/WT N2a/APP695 cell	6.25, 12.5, 25 μM	*In vitro*	Zhang et al., 2020c
	Repairment of spinosin on the learning and memory impairment induced by A*β*_25-35_ by inhibiting the inflammatory response	A*β*_25-35_	30 μmol/L	*In vitro*	Du, (2021)
Sedation effects	Increased the number of mice unclimbed the ladder and inhibited the frequency of the hole crossing	Male dd mice	200, 500, and 1,000 mg/kg	*In vivo*	Shin et al. (1978), Shin et al. (1981)
	Decrease c-Fos expression in the lateral hypothalamic area (LHA) and locus coeruleus (LC)	pathogen-free adult male mice	15 mg/kg	*In vivo*	Zhang et al., 2020a
The anxiolytic effects	Induce anxiolytic-like effects in the elevated plus maze, light/dark box test, and open field test but do not influence spontaneous activity	Male ICR mice	1.25, 2.5, and 5 mg/kg	*In vivo*	Liu et al. (2015)
Anti-melanogenic effect	Suppress *α*MSH- or UVB-induced melanogenesis in B16F10 cells without cytotoxicity	B16F10 melanoma cells	2, 5, 10, 20 μM	*In vitro*	Moon et al. (2019)
Cardioprotective effects	Weaken the myocardial tissue injury, reduce the serum levels of cTnI and LDH levels, and attenuate the apoptosis by increasing LC3B-II and reducing p62 in AMI rats	Male Wistar albino rat	5 mg/kg	*In vivo*	Gu et al. (2019)
Anti-cancer activity	ZJSP inhibited the proliferation, increased the apoptosis, and promoted the chemo-sensitivity cells *in vitro*	CRC, HCT-116, HCT-8, HCT-8FU cells	ZJSP 0, 50, 100, 150, 200 μg/ml	*In vitro*	Shan et al. (2020)
	ZJSP could reduce the CAC polyps, promote the recovery of damaged organs (heart, liver, spleen, lung, kidney, and pancreas), and raise the CRC early marker (COX-II, EMR1, and Ki67) in CAC mice	male C57BL/6J mice	ZJSP	*In vivo*	Shan et al. (2020)
			100, 200 mg/Kg		

## Pharmacokinetics

The pharmacokinetic properties are the premise of preclinical and clinical research of drugs. It provides drug toxicity and clinical application information to screen the candidate agents in the process of innovative agent development. Nowadays, HPLC (Li et al., 2014) and HPLC/MS/MS (Liu et al., 2013) were used to investigate the pharmacokinetic parameters of spinosin *in vivo* with rats (Bao et al., 2013) and dogs (Lee et al., 2020). The rat oral administration of ZJS extract (20 g/kg) containing spinosin revealed the pharmacokinetic parameters with *C*_max_ at 224 ± 82 μg/L, *T*_max_ at 5.5 ± 0.6 h, and *T*_0.5_ at 5.8 ± 0.9 h in rat plasma using the HPLC method (Li et al., 2003). The pharmacokinetic parameters of spinosin in ZJSP between the control group (NC) and insomnia model (IM) group were determined using the UPLC-MS method, and the results showed no significant differences between the two groups, but an increasing trend for CL and the decreasing trend for the AUC_0-t_ and AUC_0-∞_ of spinosin in the IM group, which indicated the faster and poorer absorption of spinosin in the IM group (Du et al., 2020). The Yin-deficiency rats were given ZJS by gavage, spinosin was detectable in the small intestine, stomach, liver, brain, large intestine, spleen, lung, heart, kidney, and blood within 10 min, and it sustained for 240 min (Guo, 2014). The distribution studies revealed that spinosin could be detected in the rat liver, spleen, kidney, lung (Li et al., 2021; Li et al., 2007), gastrointestinal (Zhang et al., 2012), bile (Liu et al., 2013), and testis (Li et al., 2007). Zhang et al. (2015) reported that spinosin could permeate the blood–brain barrier, and reach various areas of the brain such as the corpus striatum, hippocampus, cerebrum, cerebellum, and olfactory region.

Many novel dosage forms, such as solid dispersions, phospholipid complex solid dispersions and its solid lipid nanoparticles, obviously elevated solubility and bioavailability of spinosin, which effectively promoted the oral absorption of spinosin (Zhang et al., 2019; Yang et al., 2019).

The Caco-2 cell model was used to investigate the transport characteristics of spinosin, the results indicated that spinosin was transported through the intestinal mucosa *via* a passive diffusion at low concentration, while affected by P-glycoprotein (P-gp) at a high concentration with reduced absorption (Huang et al., 2016). Song et al. (2020) found the absorption mechanism of spinosin was energy-dependent monocarboxylate transporter (MCT)–mediated active transport, and the efflux process was mediated by P-gp and multidrug resistance protein (MRP), which may result in a decrease in bioavailability.

The UPLC-MS/MS was carried out to identify metabolites and evaluate the *in vivo* metabolic profile of spinosin. Three metabolites of I-phase were identified from blood and urine in depression model rats (Wang et al., 2016b). Eight I-phase metabolites of spinosin were detected in the human liver microsome incubation samples, and the cytochrome P450 enzyme was found to be the main metabolic enzyme involved in drug metabolism (Zhang et al., 2020b). Spinosin was degraded by rat intestinal bacteria *in vitro* and its metabolite was swertisin (Zhang et al., 2013). The main metabolic reactions of spinosin were decarbonylation and demethylation, hydroxylation, hydrolysis-desugar, and RDA.

**FIGURE 2:**
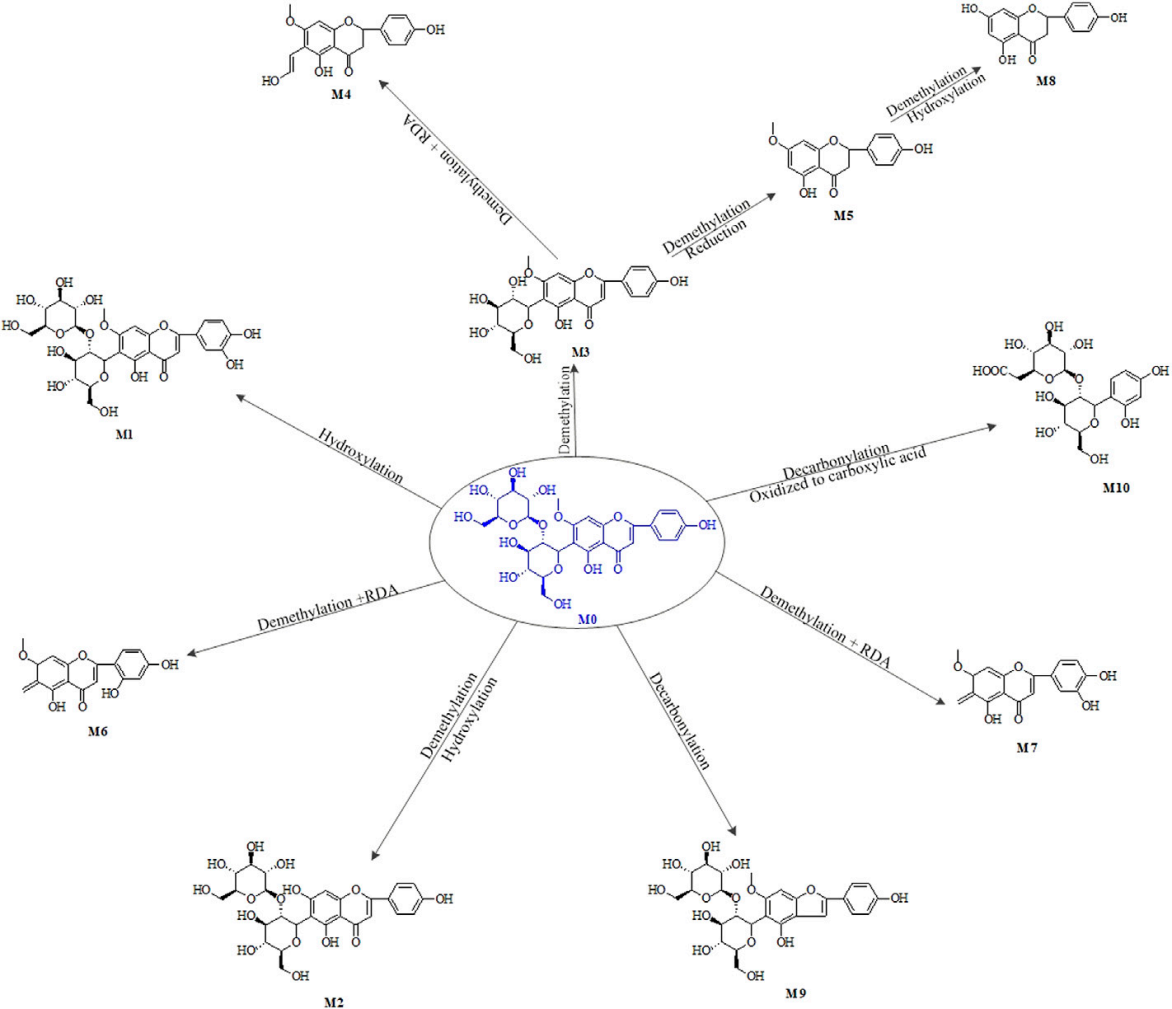
Main metabolic pathways and metabolites of spinosin.

The serum proteins, such as BSA and HSA, have the ability of binding with drugs, which play an important role in the drug metabolism. The results revealed that spinosin bound with BSA and HSA through Van der Waals force and hydrogen bond and then changed the Tyr and Trp residue microenvironments. The findings may explain the metabolism behaviors of spinosin in oxidation, intestinal hydrolysis, demethylation, and reduction (Wu et al., 2019). The pharmacokinetic studies on spinosin are shown in Table 2 and metabolites of spinosin are shown in Figure 2.

**TABLE 2:** Pharmacokinetic information of spinosin.

Model	Dose	Sample	Administration method	Quantitative method	Detail	Reference
Rat blood	20 g/kg	ZJS extract	Oral administration	HPLC	*C*_max_ = 224 ± 82 μg/L	Li et al. (2003)
					*T*_max_ = 5.5 ± 0.6 h	
					T_0.5_ = 5.8 ± 0.9 h	
Rat blood	180 mg/kg	ZJS extract	Oral administration	HPLC	*C*_max_ = 86.23 mg/L	Bao et al. (2013)
					*T*_max_ = 5.95 h	
					T_0.5_ = 5.34 h	
					AUC_0–∞_ = 269.02 mg h/L	
					CL = 0.06 L/kg/h	
					MRT = 12.15 h	
Beagle dog	200 mg/tablet	ZJS extract	Intragastric gavage	LC-MS/MS	*C*_max_ = 21.3 ng/ml	Lee et al. (2020)
					*T*_max_ = 90 min	
					T_0.5_ = 103 min	
					AUC_0–∞_ = 3,410 ng min/ml	
Rat blood	6.67 g/kg	Zaoren An-shen granule	Oral administration	HPLC	*C*_max_ = 3.452 ± 0.06 mg/L	Li et al. (2014)
					*T*_max_ = 7 ± 0.17 h	
					T_0.5_ = 2.341 ± 2.63 h	
					AUC_0–∞_ = 30.419 ± 3.58 mg h/L	
					CL = 0.589 ± 0.08 L/kg/h	
SD rat	6.8 g/kg	ZJS extract	Intragastric gavage	UHPLC-Q-Orbitrap-MS	*C*_max_ = 40.08 mg/L	Du et al. (2020)
					*T*_max_ = 0.23 h	
					T_0.5_ = 2.75 h	
					AUC_0–∞_ = 44.65 *μ*g h/L	
					CL = 1,099.25 L/kg/h	
SD rat	5 mg/kg	Spinosin	Femoral vein injection	HPLC-MS	Blood: *C*_max_ = 5.59 ± 2.65; AUC_0–∞_ = 205.70 ± 80.79 mg min/L; T_1/2_ = 48.07 ± 4.71; CL = 3.00 × 10^–2^ ± 5.10×10^–3^;V = 2.08 ± 0.35 L/kg	Ma et al. (2012)
					Bile: *C*_max_ = 695.40 ± 162.90; AUC_0–∞_ = 7.77 × 10^–4^ ± 2.13×10^–4^ mg min/L; T_1/2_ = 97.20 ± 37.63; CL = 1.00 × 10^–2^ ± 2.00×10^–3^;V = 9.40 × 10^–2^ ± 1.80×10^–2^ L/kg	
					Brain: *C*_max_ = 5.10 × 10^–2^ ± 7.5×10^–2^; AUC_0–∞_ = 2.09 ± 0.03 mg min/L; T_1/2_ = 42.18 ± 13.71; CL = 1.72 ± 0.28; V = 101.67 ± 16.45 L/kg	
Rat	10, 20, 40 mg/L	Spinosin	Oral administration	HPLC	Spinosin was absorbed in all segments gastrointestinal in the pattern of first-order kinetics with the passive diffusion absorption mechanism	Zhang et al. (2012)
SD rat	5 mg/kg	Spinosin	Intravenous administration	UPLC-MS/MS	Spinosin could permeate the blood–brain barrier, and reached the various areas of the brain such as the corpus striatum, hippocampus, cerebrum, cerebellum, and olfactory region	Zhang et al. (2015)
Wistar rat	20 mg/kg	Spinosin	Intravenous administration	HPLC	Spinosin was detected in the liver, brain, spleen, and kidney	Li et al. (2007)
					T_0.5_ = 6.66 h	
					AUC_0–∞_ = 2.83 mg h/L	
					CL = 1.42 L/kg/h	
Rat bile	9 g/kg	Shensong Yangxin capsules	Oral administration	UPLC-MS/MS	Total bile excretion of the original drug in 24 h accounted for 1.096%	Liu et al. (2013)
SD rats	1.0 g/ml	Zaoren Anshen prescription	Intragastric gavage	UPLC-MS/MS	Spinosin mainly distributed in the kidney, liver, heart, spleen, lung, and brain	Li et al. (2021)
Rat blood	20 mg/kg	Spinosin	Oral administration	HPLC	Solid dispersions and phospholipid complex solid dispersions can promote the oral absorption of spinosin	Zhang et al. (2019)
SD rat blood	20 mg/kg	Spinosin	Oral administration	HPLC	Compared with spinosin, the relative bioavailability and oral absorption of the phospholipid complex and phospholipid complex solid lipid nanoparticles increased	Yang et al. (2019)
Caco-2 cell	2, 10, 40, and 80 μg/ml	Spinosin	—	UPLC-MS/MS	Spinosin is transported through the intestinal mucosa via a passive diffusion at a low concentration while affected by P-gp at a high concentration with reduced absorption	Huang et al. (2016)
Caco-2 cell	10, 20, 50, 100, 200, and 400 μmol/L	Spinosin	—	UPLC-MS/MS	Absorption mechanism of spinosin was energy-dependent MCT-mediated active transport while the efflux process of spinosin was mediated by P-gp and MRP.	Song et al. (2020)
Rat blood	4.97 mg/L	Shaozao capsules	Oral administration	UPLC-Q-TOF-MSE	Three spinosin metabolites were detected in rat blood	Wang et al., 2016b
Human liver microsome	200 µM	Spinosin	—	UPLC-Q-TOF-MS	Eight spinosin metabolites were detected in human liver microsome incubation samples	Zhang et al., 2020b
SD rat intestinal flora	5, 10, 20, 40 mg/ml	Spinosin	—	HPLC-MS/MS	Spinosin can be metabolized *in vitro* by rat intestinal bacteria and its metabolite was swertisin	Zhang et al. (2013)

## Toxicity

The toxicity of spinosin was studied by intraperitoneally injecting into mice with graded doses ranging from 200 mg/kg to 10 g/kg and there was no mortality of mice even at the highest dosage, which suggested the safety of spinosin (Shin et al., 1978).

Spinosin also did not exhibit cytotoxicity on HaCat, B16F10, and Hs27 cells at 20 μM (Moon et al., 2019). Spinosin had cytotoxicity in N2a/APP695 cells at 200 and 400 μM, but no cytotoxicity in N2a/WT cells at 400 μM (Zhang et al., 2020c). The cytochrome P450 participated in the drug metabolism process and mediated the drug–herb interactions, which have attracted much attention in recent years. The activation effects between CYP450 and drugs may increase the risk of drug application. One report indicated that spinosin exhibited inactive effects on CYP3A4 in human liver microsomes (Bai et al., 2020).

Spinosin, the main active *C*-glycoside constituent from ZJS, is highly consistent with pharmacological and toxicological properties of ZJS. The ZJS decoction was administered to mice at 15 g/kg by gavage, and no toxicity was observed in 48 h (Shen, 2011). The administration routes affected the drug’s toxicity. ZJS decoction and ethanolic extract of ZJS were injected intravenously into mice and the LD_50_ values were determined as 14.3 ± 20.0 and 27.5 ± 2.4 g/kg, respectively. The same samples were orally administered to mice at the dosage of 340 g/kg, but no case of mortality was observed (Wang et al., 2009). The chickens were orally administered with ZJS solution at the dosages ranging between 2.5 and 20 g/(kg d) and the maximum tolerable dosage of ZJS solution was calculated as more than 20 g/(kg d), while the LD_50_ was not determined. The experiments suggested that ZJS solution had no acute toxicity and no long-time toxicity (Li et al., 2010).

## NMR phenomenon

The interesting NMR phenomenon of spinosin was observed in 2000 (Gong et al., 2000). Both 1H and 13C NMR data of spinosin exhibited the partial carbons and protons signal splits appearance at room temperature (298 K), and doublet signals disappeared as the temperature rose to coalescence temperature (*T*_*c*_ 363 K). The results of the variable-temperature experiments suggested the presence of two rotational isomerisms at room temperature. Compared to the NMR data with compounds that have similar structures, only the constituents with 7-OCH_3_ in the flavone-6-C-glycoside skeleton exhibited the aforementioned NMR signal pattern (Gong et al., 2000). The variable-temperature 1H NMR experiments explained that the high energy barrier about the *C*-6-*C*-1″ bonds prevents the interchange between rotamers at room temperature. The theoretical (MM2) calculations revealed the minimum energy of two conformations (energy difference ca 0.84 kJ/mol) and the separated energy barrier of ca 67 kJ/mol (Lewis et al., 2000). The finds were approved by many reports in analogs of spinosin, such as 6‴-feruloyl-spinosin, 6‴-acetyl-spinosin, and isovitexin-2″-*O*-arabinoside (Song et al., 2020; Zhou and Yan, 2021). The aforementioned NMR signal features can be used to quickly distinguish the chemical skeleton and analyze the structure of substituent position. The 1D NMR spectra of spinosin at 298 and 387 K are shown in Figure S1A-B.

## Conclusion

This article reviewed the bioactivities and the mechanisms of spinosin (Table 1), its pharmacokinetics parameters (Table 2) and security, as well as characteristic NMR performance. However, many issues need to be further illustrated in further studies. First, a few reports on pharmacological activities and the reported bioactivities of spinosin mainly focused on the phenotypic aspect, but there is lack of an in-depth specific interpretation on the mechanism research. Therefore, it is extremely meaningful to explore the molecular mechanism of its biological activities. Second, the little existing evidence suggested the safety of spinosin *in vitro* and *in vivo*, but it is difficult to fully evaluate its security due to lack of research evidence. Hence, it is necessary to systematically evaluate its safety and toxicity *in vitro* and *in vivo* for the clinical application. Third, mice are also widely used for pharmacokinetic studies but there have been no relevant reported pharmacokinetic studies on spinosin. So, the comprehensive pharmacokinetic research about spinosin and ZJS in rodent models should be conducted in future.

## Glossary

5-HTP: 5-hydroxytryptophan
AD: Alzheimer’s disease
AMI: acute myocardial infarction
APP: amyloid precursor protein
A*β*_1-42_: amyloid-*β*_1-42_
BDNF: brain-derived neurotrophic factor
BSA: bovine serum albumin
CAC: colitis-associated cancer
ChAT: choline acetyltransferase
CL: clearance
CRC: colorectal cancer
cTnI: cardiac troponin I
GABA_A_: *γ*-aminobutyric acid A
GSK3*β*: glycogen synthase kinase-3*β*
HPLC: high-performance liquid chromatography
HSA: human serum albumin
IM: insomnia model
LC: locus coeruleus
LC3B-II: microtubule-associated protein one light chain 3B-Ⅱ
LDH: lactate dehydrogenase
LHA: lateral hypothalamic area
LORR: loss of righting reflex
LTP: long-term potentiation
MCT: monocarboxylate transporter
MDA: malondialdehyde
MRP: multidrug resistance protein
NC: normal control
NMR: nuclear magnetic resonance
NREM: non-rapid eye movement
PCPA: p-chlorophenylalanine
P-gp: P-glycoprotein
PK: pharmacokinetic
RDA: Retro Diels–Alder reaction
REM: rapid eye movement
ROS: reactive oxygen species
SWS: slow-wave sleep
TCM: traditional Chinese medicine
ZJS: *Ziziphus jujuba* Mill. var. *spinosa*
ZJSP: polyphenols of *Ziziphus jujuba* Mill. var. *spinosa*
