# Supplementary figures and images for: The pharmacology, pharmacokinetics, and toxicity of spinosin: A mini review
Source: Front Pharmacol. 2022 Sep 12;13:938395. doi: 10.3389/fphar.2022.938395 (PMC9525219; doi:10.3389/fphar.2022.938395)

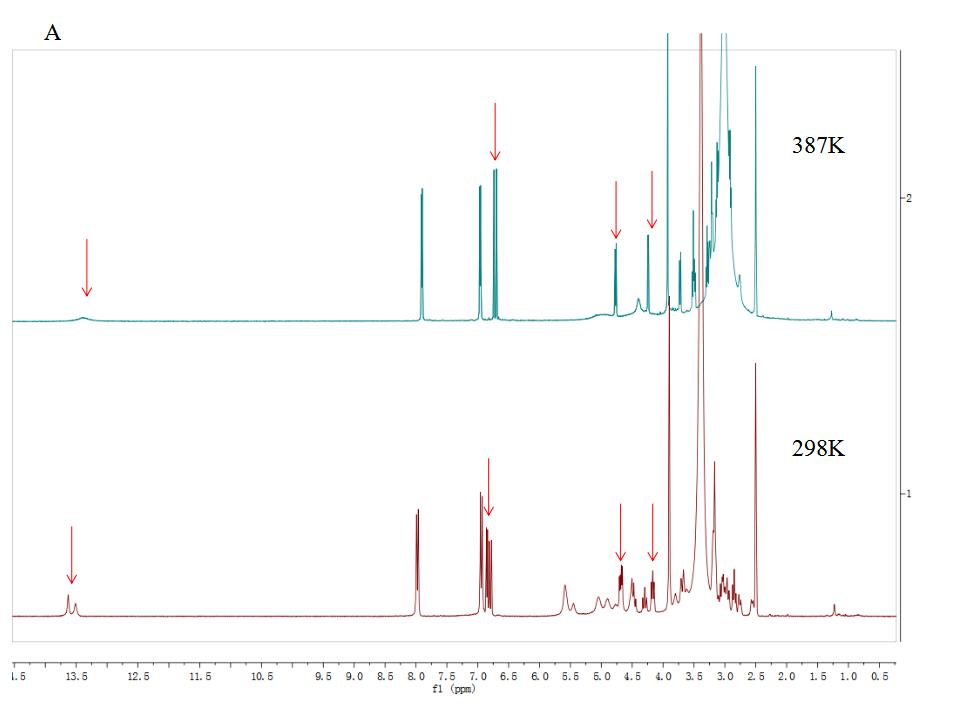

Supplement: Supplementary file 1 [file Image1.JPEG]

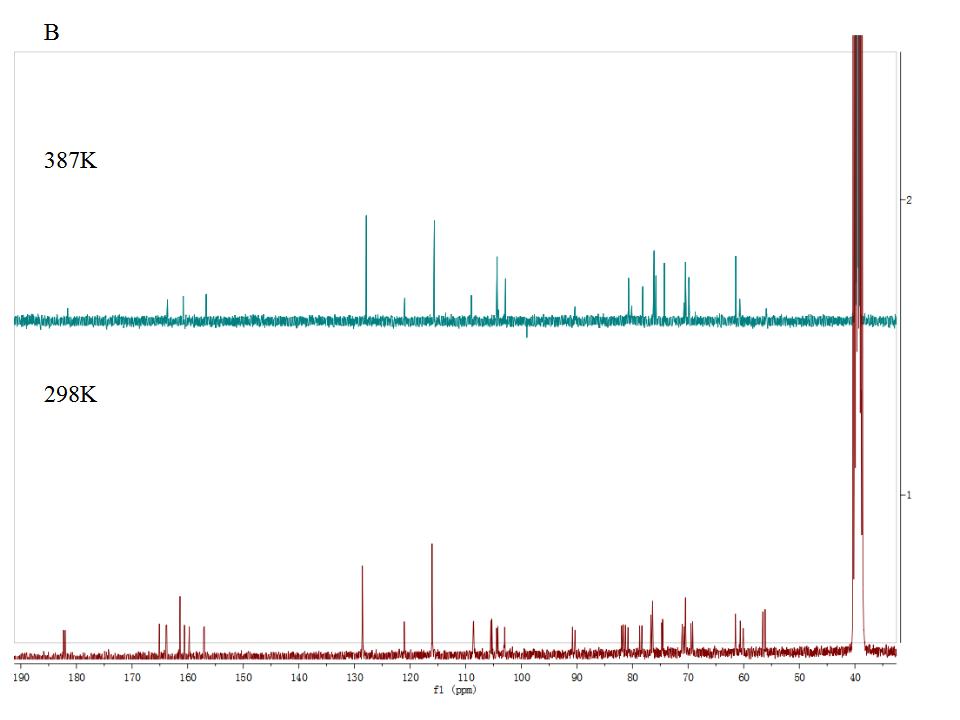

Supplement: Supplementary file 2 [file Image2.JPEG]
